# Glutamine metabolism-related genes predict the prognostic risk of acute myeloid leukemia and stratify patients by subtype analysis

**DOI:** 10.1186/s41065-024-00338-8

**Published:** 2024-09-19

**Authors:** Jie Zhou, Na Zhang, Yan Zuo, Feng Xu, Lihua Cheng, Yuanyuan Fu, Fudong Yang, Min Shu, Mi Zhou, Wenting Zou, Shengming Zhang

**Affiliations:** 1 Department of Hematology, Deyang People’s Hospital, No. 173 Taishan North Road, Section 1, Jingyang District, Deyang, 618000 Sichuan China; 2 Department of health management, Guangdong Second Provincial General Hospital, Guangzhou, 510317 Guangdong China

**Keywords:** Acute myeloid leukemia, Glutamine, Prognostic model, Molecular subtype, Immune infiltration

## Abstract

**Background:**

Acute myeloid leukemia (AML) is a genetically heterogeneous disease in which glutamine (Gln) contributes to AML progression. Therefore, this study aimed to identify potential prognostic biomarkers for AML based on Gln metabolism-related genes.

**Methods:**

Gln-related genes that were differentially expressed between Cancer Genome Atlas-based AML and normal samples were analyzed using the limma package. Univariate, least absolute shrinkage, selection operators, and stepwise Cox regression analyses were used to identify prognostic signatures. Risk score-based prognostic and nomogram models were constructed to predict the prognostic risk of AML. Subsequently, consistent cluster analysis was performed to stratify patients into different subtypes, and subtype-related module genes were screened using weighted gene co-expression network analysis.

**Results:**

Through a series of regression analyses, *HGF*, *ANGPTL3*, *MB*, *F2*, *CALR*, *EIF4EBP1*, *EPHX1*, and *PDHA1* were identified as potential prognostic biomarkers of AML. Prognostic and nomogram models constructed based on these genes could significantly differentiate between high- and low-risk AML with high predictive accuracy. The eight-signature also stratified patients with AML into two subtypes, among which Cluster 2 was prone to a high risk of AML prognosis. These two clusters exhibited different immune profiles. Of the subtype-related module genes, the HOXA and HOXB family genes may be genetic features of AML subtypes.

**Conclusion:**

Eight Gln metabolism-related genes were identified as potential biomarkers of AML to predict prognostic risk. The molecular subtypes clustered by these genes enabled prognostic risk stratification.

## Highlights

- Eight genes were identified as potential prognostic biomarkers of acute myeloid leukemia (AML).
- The prognostic and nomogram models can accurately predict the AML prognostic risks.
- Eight prognostic signatures stratified AML patients into two subtypes with different prognostic patterns and immune profiles.
- *HOXA* and *HOXB* family genes may be genetic features of AML subtypes.

## Background

Acute myeloid leukemia (AML) is a fatal cancer characterized by increased self-renewal and uncontrolled proliferation of malignant bone marrow stem cells, accompanied by infection, hemorrhage, and organ infiltration [1]. A small percentage of cases have been determined to be affected by causative factors such as chemotherapy or chemical exposure, but the vast majority develop due to chromosomal abnormalities and gene mutations [2]. As a genetically heterogeneous disease, more than 97% of AML cases have recognizable somatic mutations [3]. Therefore, cytogenetic markers are currently the most important indicators for the risk stratification and treatment of patients with AML [4]. However, AML is still associated with relatively poor survival, and recent data have reported a 5-year overall survival rate of 21% for AML, similar to that of solid organ malignancies with a high fatality rate [5]. This difficulty lies in the fact that the prognosis of AML is closely related to the genetic characteristics of the disease, leading to variability in treatment and prognosis. This study summarized a series of gene mutations, including tumor protein p53 (*TP53*), nucleophosmin, fms-related receptor tyrosine kinase 3, and CCAAT enhancer binding protein alpha, that may serve as potential prognostic markers and targets for AML [6, 7]. However, the complexity and specificity of each patient’s genetic profile have forced researchers to continually identify novel prognostic markers to predict an individual’s response to treatment, thereby enabling effective personalized treatment.

Metabolic reprogramming is a key manifestation of AML and is closely associated with clinical diagnosis, risk stratification, and targeted drug development [8]. Cellular metabolism in AML is genotype-specific and is accompanied by epigenetic changes, somatic mutations, and activation of downstream cancer-promoting pathways [9]. Amino acid metabolism plays a role in regulating redox homeostasis and maintaining cell proliferation [10]. Glutamine (Gln), a non-essential amino acid, is the most abundant amino acid in human blood. However, when the energy requirement for the rapid proliferation of cancer cells is not met, Gln can be converted to be conditionally essential and contribute to AML cell proliferation [11]. Removal of Gln induces apoptosis in AML cells by inhibiting the mechanistic target of rapamycin complex 1 pathway [12]. Therefore, screening for molecular targets of Gln metabolism may help develop novel AML treatment strategies and improve patient prognosis.

However, no systematic study has been conducted to comprehensively screen biomarkers of AML from the perspective of Gln metabolism to predict prognosis. Therefore, we conducted a series of bioinformatics analyses to screen prognostic signatures from Gln metabolism-related genes to predict prognostic risk and stratify patients to further identify their genetic and immune characteristics. The potential prognostic markers identified in this study may help optimize treatment choices for patients, reduce prognostic risk, and deepen the biological understanding of AML.

## Methods

### Data search and information

Gene expression profiles of AML were obtained from the Cancer Genome Atlas (TCGA) [13]. Based on the available clinical information, AML samples with prognostic information and survival time over 30 days, totaling 149 cases, were enrolled in this study. In addition, 337 whole blood samples from the Genotype-Tissue Expression (GTEx) database were used as normal controls. These 486 samples were used as the training set for subsequent analyses.

The validation set, GSE71014, was obtained from the Gene Expression Omnibus (GEO) database [14]. It was detected on the GPL10558 Illumina HumanHT-12 V4.0 expression bead chip and comprised 104 AML samples. Finally, 96 AML samples with a survival time of more than 30 days were included in this study.

### Screening of differentially expressed genes (DEGs) related to gln metabolism

In GeneCards, 704 protein-coding genes with correlation coefficients greater than eight for Gln metabolism were defined as Gln-related genes, of which 639 were matched to the training set. By comparing the gene expression profiles of these 639 genes between AML and normal groups, Gln-related DEGs (Gln-DEGs) were selected using the limma package (Version 3.52.4) [15], at thresholds of adj.*p* < 0.05 & |log_2_fold change (FC)| > 2.

### Genetic mutation of Gln-DEGs

The somatic mutation maf files of AML, processed using Mutect software, were downloaded from TCGA. The oncoplot function of the R package maftools was used to plot the mutation waterfall of the TOP10 mutated genes in the Gln-DEGs.

### Identification of prognostic signatures to construct the risk score-based model

Based on the Gln-DEGs expression matrix, univariate Cox regression analysis in the R survival package was employed to screen for genes significantly correlated with prognosis at the expression level with a cutoff of *p* < 0.01. The least absolute shrinkage and selection operator (LASSO) Cox regression analysis in the R. glmnet package (version 1.2) [16] was used to further screen key genes by penalization parameter tuning through 10-fold cross-validation. Finally, the prognostic signatures were screened using stepwise Cox regression analysis in the R. survminer package (version 0.4.9) to construct a prognostic model according to the risk score, which was calculated as follows:

$${\text{risk score}} = \exp(\upbeta 1 \times 1 + \upbeta 2 \times + \cdots + \upbeta {\text{n X n}})$$

In this formula, exp indicates the expression level of prognostic signatures, while β represents the stepwise regression coefficient of this gene. The high- and low-risk groups were bounded by the median risk score in the training and validation cohorts. The association between risk score and the actual prognosis was assessed using Kaplan-Meier (KM) analysis in the survival package of R3.6.1 (version 2.41-1). In contrast, the predictive efficacy of the risk score for prognosis at 1, 3, and 5 years was estimated using receiver operating characteristic (ROC) curves.

### Screening of independent prognostic factors to establish a nomogram model

This study further included clinical information (age, race, sex, and FAB subtype) as well as risk scores in the univariate and multivariate Cox regression analyses to screen for independent prognostic factors with a *p*-value less than 0.05. These factors were used to construct a nomogram model to evaluate the predictive relationship between these factors and prognosis using the rms package in R (version 5.1-2) [17]. KM and ROC curves were used to validate the predictive efficacy of the nomogram.

### Immuno-analysis of prognostic signatures

The CIBERSORT algorithm [18] was used to estimate the infiltration abundance of key immune cells in high- and low-risk groups. The cor function in R was used to evaluate the relationship between the prognostic signatures and immune cells by calculating the Spearman correlation coefficient. Expression data for 36 immune checkpoint genes and 15 human leukocyte antigen (HLA) family genes were also extracted to compare their expression differences between high- and low-risk groups using the Wilcoxon test.

### Identification and comparison of molecular subtypes of AML

In this study, we used the R. ConsensusClusterPlus package (Version:1.58.0) [19] to perform a consistent clustering analysis of AML samples using hierarchical clustering based on Spearman correlation coefficients to cluster and identify different AML subtypes. Similarly, the KM curve was used to compare the survival differences between the two risk groups. The abundance of immune cell infiltration in each subtype sample was calculated using CIBERSORT and was compared between the two subtypes.

### Screening of key genes related to AML subtypes using weighted gene co-expression network analysis (WGCNA)

This study performed WGCNA on the top 20% of genes with an absolute deviation from the median of expression value screening in AML samples. WGCNA utilizes module eigengenes to differentiate modules. By calculating the correlations between modules and modules as well as between modules and traits, modules that were highly correlated with the traits were screened, and key genes were selected from the modules. This study used subtypes as phenotypic traits, and key genes were selected from modules related to AML subtypes using the WGCNA package in R (version 1.71) [20].

### Protein-to-protein interaction (PPI) analysis

The STRING database (version 11.5) [21] was used to predict the interactions between gene-encoded proteins. In this study, PPI analysis was carried out on subtype-related module genes based on the STRING database, with the species as homo sapiens and the parameter set as an interaction score of 0.4.

## Results

### Mutational analysis on Gln-DEGs

**Fig. 1:**
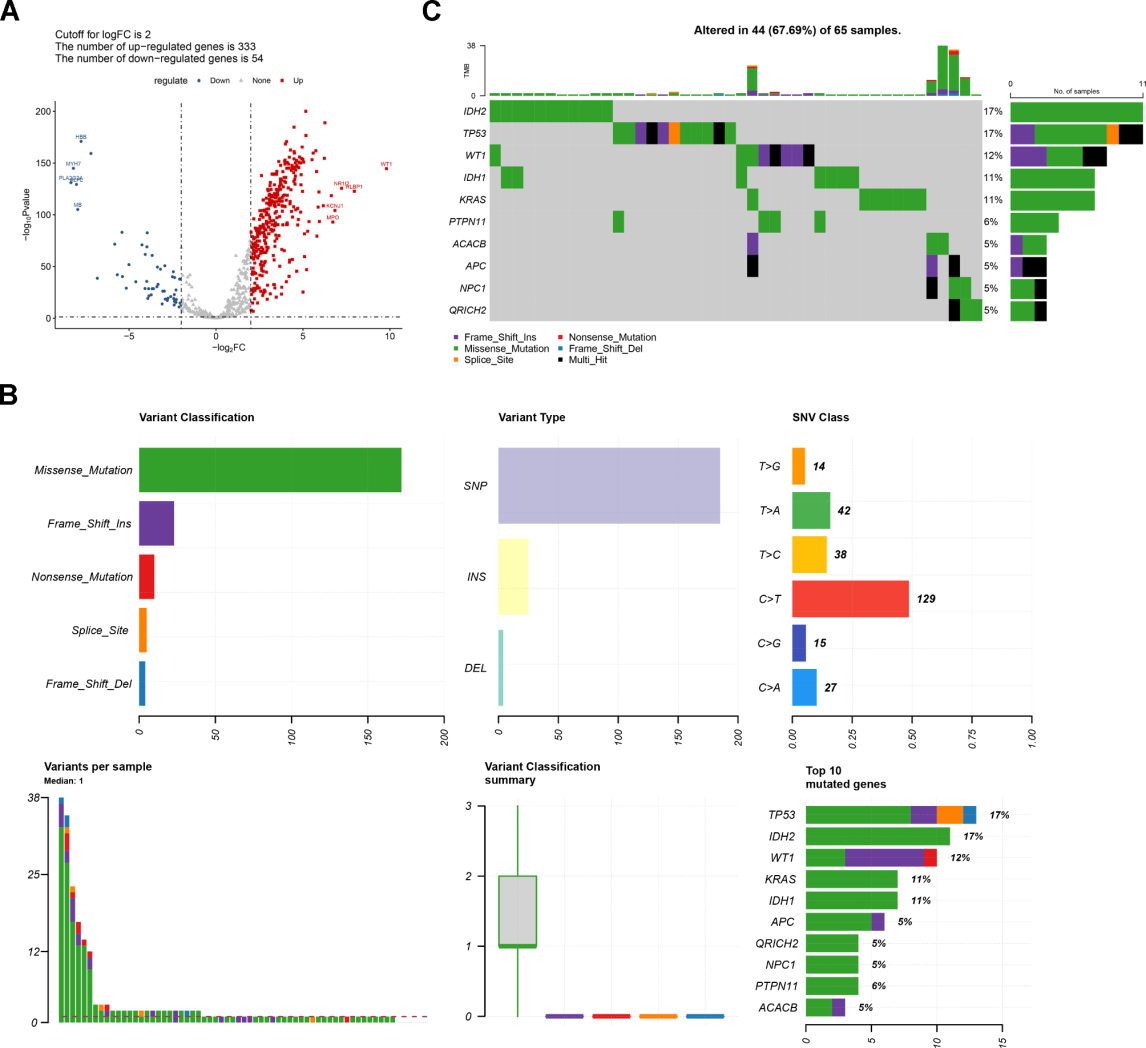
**Analysis of somatic variants in Gln-DEGs**. **A**. The volcano plot showed 387 Gln-DEGs between AML and normal samples. **B**. The summary plot showed information on the variant classification, variant types, variant numbers in each sample, and the top10 mutated genes. **C**. Waterfall plot depicted the top10 mutated Gln-DEGs in terms of tumor mutation burden. *IDH*, isocitrate dehydrogenase; *TP53*: tumor protein p53; *WT1*: transcription factorWT1; *IDH1*, isocitrate dehydrogenase (NADP(+)) 1; *KRAS*, KRAS proto-oncogene; *PTPN11*, protein tyrosine phosphatase non-receptor type 11; *ACACB*: acetyl-CoA carboxylase beta; *APC*, *APC* regulator of WNT signaling pathway; *NPC1*, NPC intracellular cholesterol transporter 1; *QRICH2*: glutamine rich 2

By comparing the expression data of 639 Gln-related genes in AML and normal samples, 387 Gln-DEGs were identified (Fig. 1A). Genetic level-based analyses suggested that most somatic variants in these genes were missense mutations (Fig. 1B). Among these, the TOP10 genes with the highest mutation frequencies in AML included *IDH*, *TP53*, *WT1*, *IDH1*, *KRAS*, *PTPN11*, *ACACB*, *APC*, *NPC1*, and *QRICH2* (Fig. 1C).

### Identification of prognostic signatures from Gln-DEGs

**Fig. 2:**
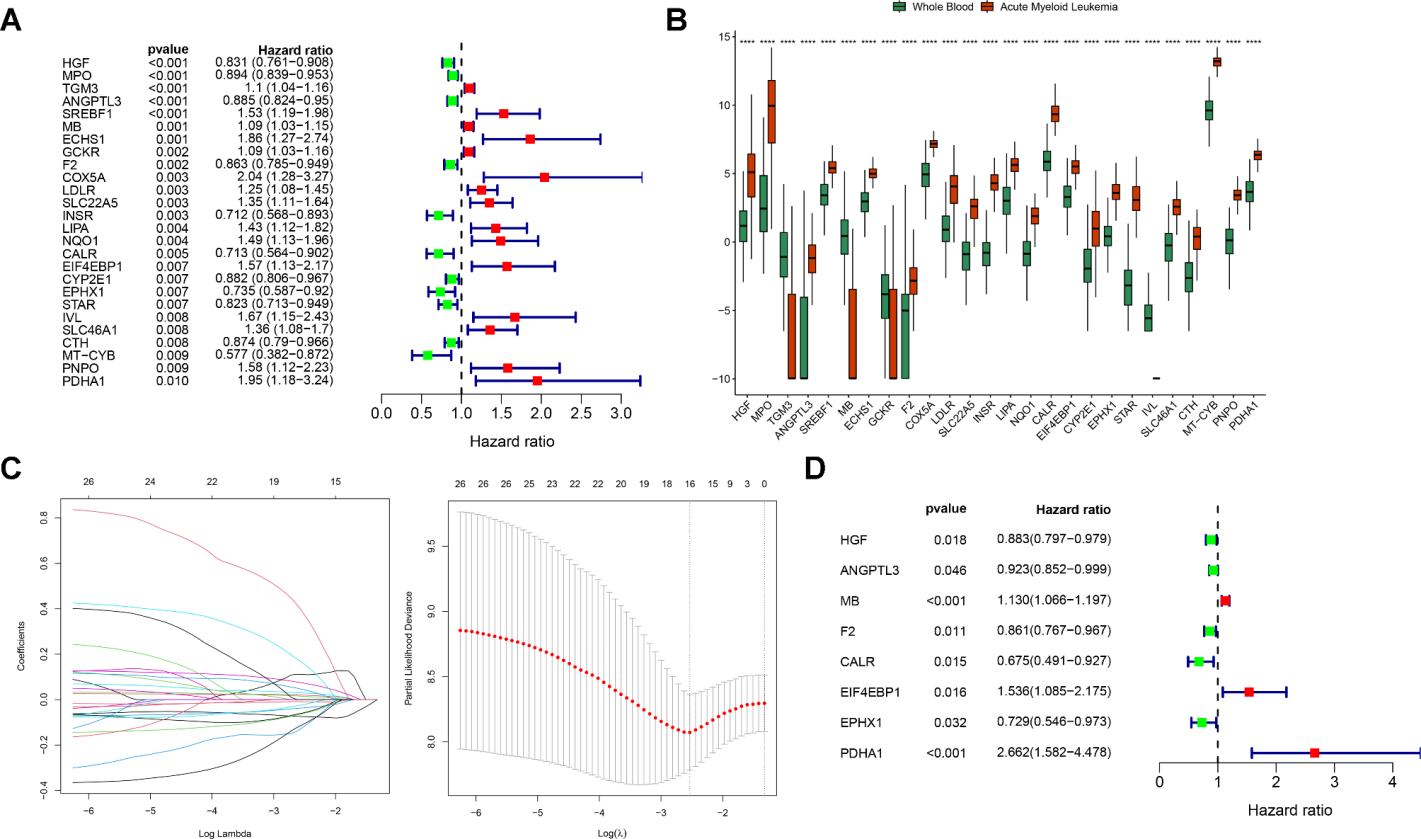
**Screening of prognostic signatures using the univariate**,** LASSO**,** and stepwise Cox regression analyses**. **A**. The univariate Cox analysis of 26 Gln-DEGs. **B**. Differences in gene Expression between AML and whole blood samples. ****p < 0.0001. **C**. Left panel: Distribution of the LASSO coefficients. Right panel: Selection of lambda min based on the likelihood deviation of the LASSO coefficient distribution. **D**. Eight prognostic signatures identified using stepwise Cox regression analysis

Of the 387 Gln-DEGs, 26 genes significantly correlated with prognosis were screened using univariate Cox regression analysis (Fig. 2A). The expression of these genes was significantly different between AML and whole blood samples (*p* < 0.0001) (Fig. 2B). Then, the optimal gene list comprising 16 genes was selected from the LASSO analysis (Fig. 2C), followed by the further identification of eight prognostic signatures (hepatocyte growth factor (*HGF*), angiopoietin-like 3 (*ANGPTL3*), myoglobin (*MB*), coagulation factor II (*F2*), calreticulin (*CALR*), eukaryotic translation initiation factor 4E binding protein 1 (*EIF4EBP1*), epoxide hydrolase 1 (*EPHX1*), and pyruvate dehydrogenase E1 subunit alpha 1 (*PDHA1*)) using the stepwise Cox regression analysis (Fig. 2D).

### Construct a risk score-based model to predict prognostic risks

**Fig. 3:**
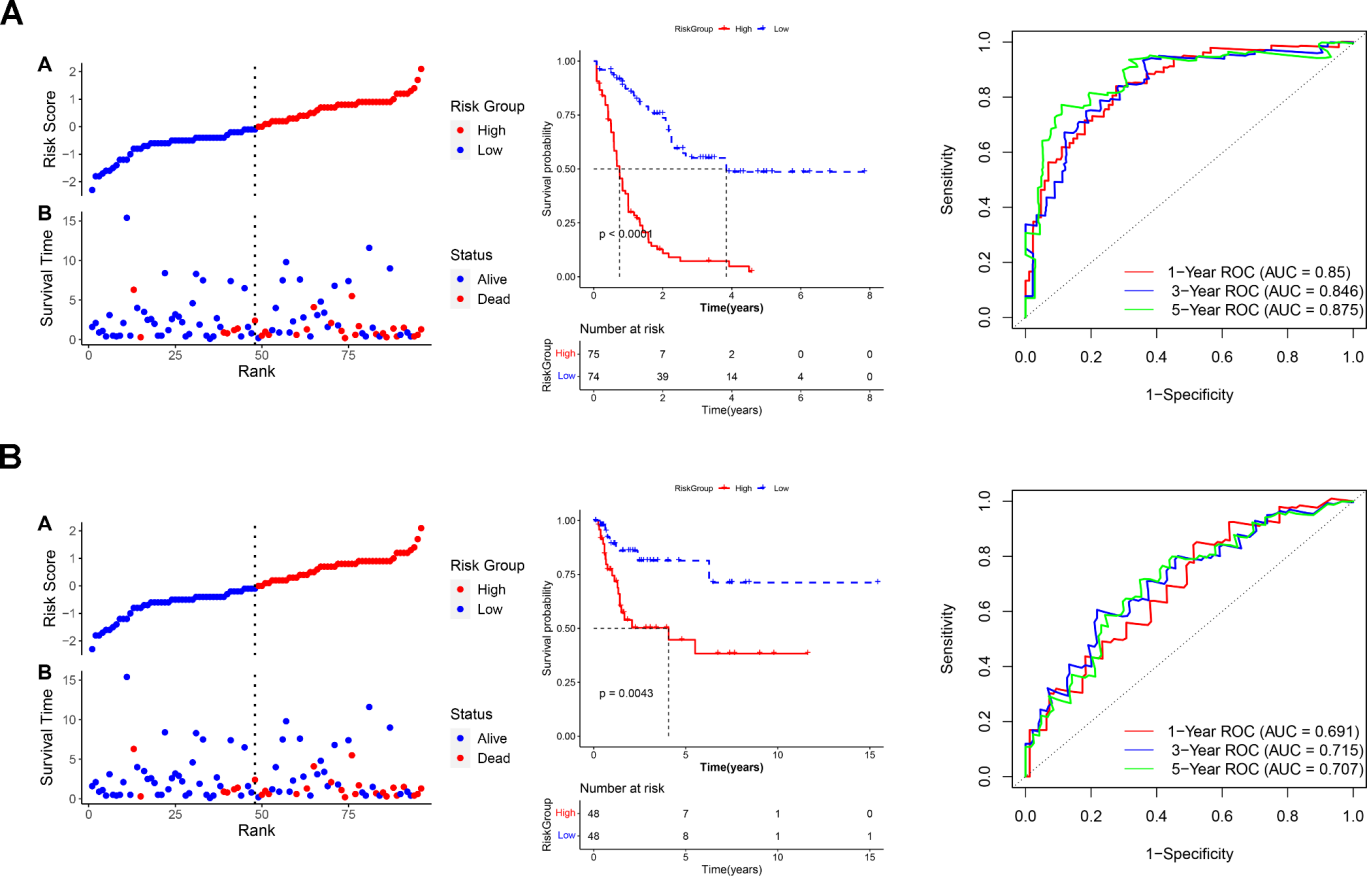
**Construction a risk score-based model in the training set (A) and the verification of the model in the validation cohort (B)**. Left panel: risk score distribution and survival status of all AML samples; Middle panel: KM curve displaying the difference in survival probability between high- and low-risk groups; Right panel: ROC curves showing the ability of the model to predict 1-, 3-, and 5-year survival prognosis

Based on the expression data and stepwise regression coefficients of the eight prognostic signatures, the risk score for each sample in the training and validation sets was calculated to establish the prognostic models. After defining the high- and low-risk groups in the training set, it was suggested that patients who died were more frequently distributed in the group with a high prognostic risk (Fig. 3A). The KM curves also confirmed a significant difference in survival probability between the two groups (Fig. 3A). ROC curves were then plotted to assess the sensitivity and specificity of the model based on the training set in predicting AML prognostic risks. The areas under the curve (AUCs) of 1-, 3-, and 5-year ROC were 0.85, 0.846, and 0.875, respectively (Fig. 3A), suggesting the predictive potential of this prognostic model. Furthermore, the model was reconstructed using the validation set, and the results were consistent with the above findings. The validation set-based prognostic model also significantly distinguished the survival status of samples under different risk groups and accurately predicted AML prognostic risks (Fig. 3B).

### Screening for independent prognostic factors and constructing a nomogram to predict survival

**Fig. 4:**
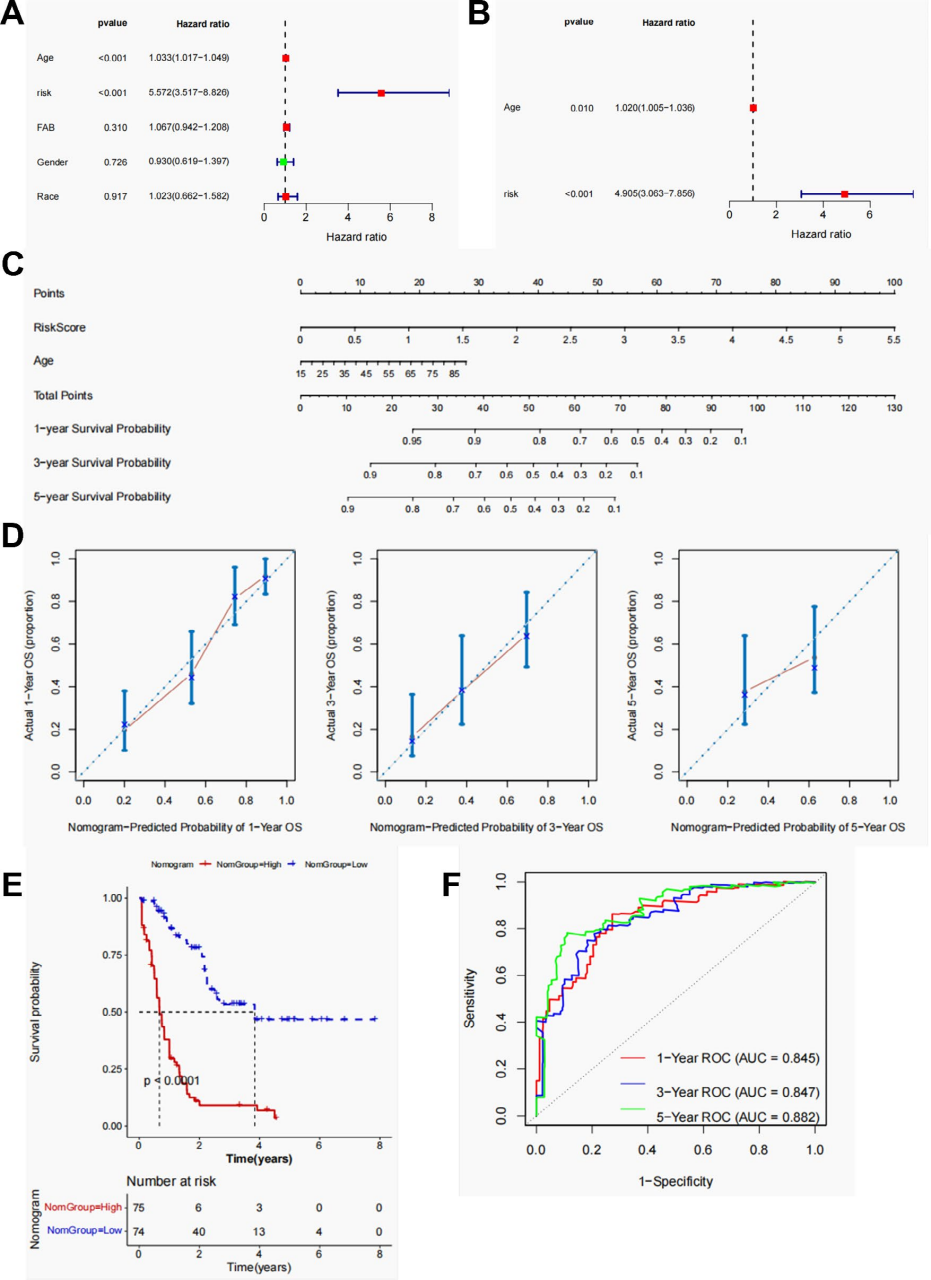
**Screening of independent prognostic factors using the univariate and multivariate Cox regression analyses and the construction of the nomogram model to predict prognosis**. **A**: Forest maps showed independent prognostic factor screening by univariate Cox regression analysis. **B**. Forest maps showed independent prognostic factor screening using multivariate Cox regression analyses. **C**: Age and risk scores were used to create a nomogram model for predicting survival. **D**. The fitness of the model predicted the overall survival to actual survival. **E**. The KM curve showed the survival difference between samples with high- and low-risk groupings using the nomogram model. **F**. ROC curves confirmed the power of the nomogram model in predicting the 1-, 3-, and 5- survival statuses

By integrating the clinical information and risk scores, independent prognostic factors were selected using univariate and multivariate Cox regression analyses. Age (HR = 1.020, 95% Cl = 1.005–1.036, *p* = 0.010) and risk score (HR = 4.905, 95% Cl = 3.063–7.856, *p* < 0.001) were determined to have prognostic independence (Fig. 4A, B). These two factors were incorporated into the nomogram model to predict survival (Fig. 4C). The fitting curves suggested that the overall survial predicted by the model converged with the actual survival (Fig. 4D). The KM curve further demonstrated that patients with higher risk scores had significantly worse prognoses (Fig. 4E). The AUCs of the ROC curves were all over 0.84, indicating that the nomogram was highly sensitive and specific in predicting the 1-, 3-, and 5- survival statuses (Fig. 4F).

### Evaluation of immune landscape

**Fig. 5:**
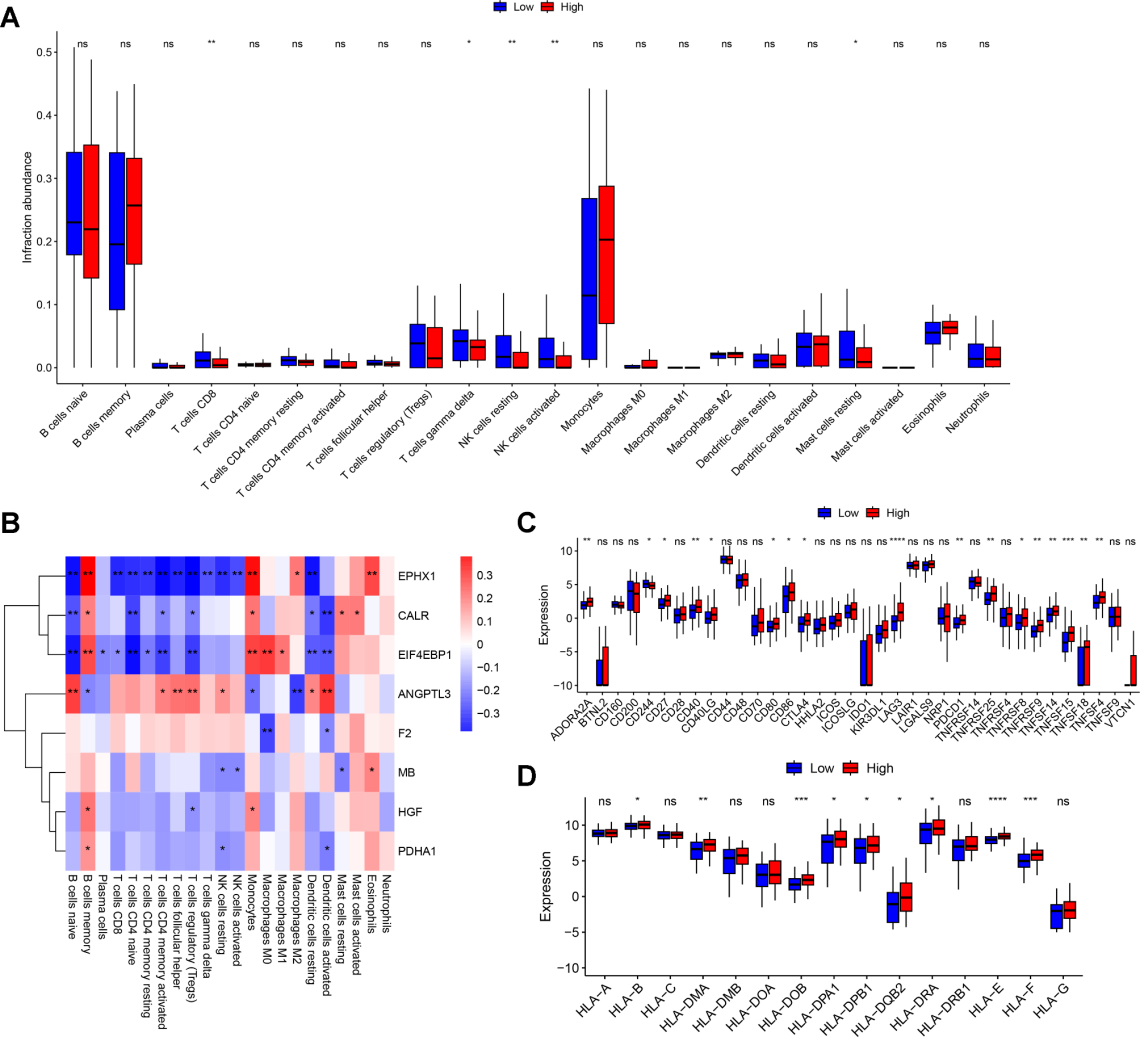
**Comparison of infiltration of immune cells and expression of immune-related genes between the high- and low-risk groups**. **A** Differences in the infiltration of 22 types of immune cells between the high- and low-risk groups. **B**: Correlation between immune cell infiltration and expression of eight prognostic signatures. **C** Box plot showed differences in the expression of immune checkpoint genes between the two groups. *p < 0.5; **p < 0.01; ***p < 0.001; ****p < 0.0001. **D**. The box plot presented the expression differences in HLA family genes between the two groups. *p < 0.5; **p < 0.01; ***p < 0.001; ****p < 0.0001

In this study, the CIBERSORT algorithm was used to quantify immune cells in all samples. By comparing the high- and low-risk groups, we found five types of immune cells (CD8 T cells, γδT cells, resting NK cells, activated NK cells, and resting mast cells) were significantly different infiltrated between the two groups, and the proportions of these five cells were all significantly decreased in the high-risk group (Fig. 5A). The correlation between the expression levels of the eight prognostic signatures and the infiltration levels of these cells was shown in Fig. 5B. Furthermore, the majority of immune checkpoint genes and HLA family genes were found to have significant differences in expression between the high- and low-risk groups (Fig. 5C and D), suggesting different immune statuses between the two groups.

### Identification and comparison of AML molecular subtypes

**Fig. 6:**
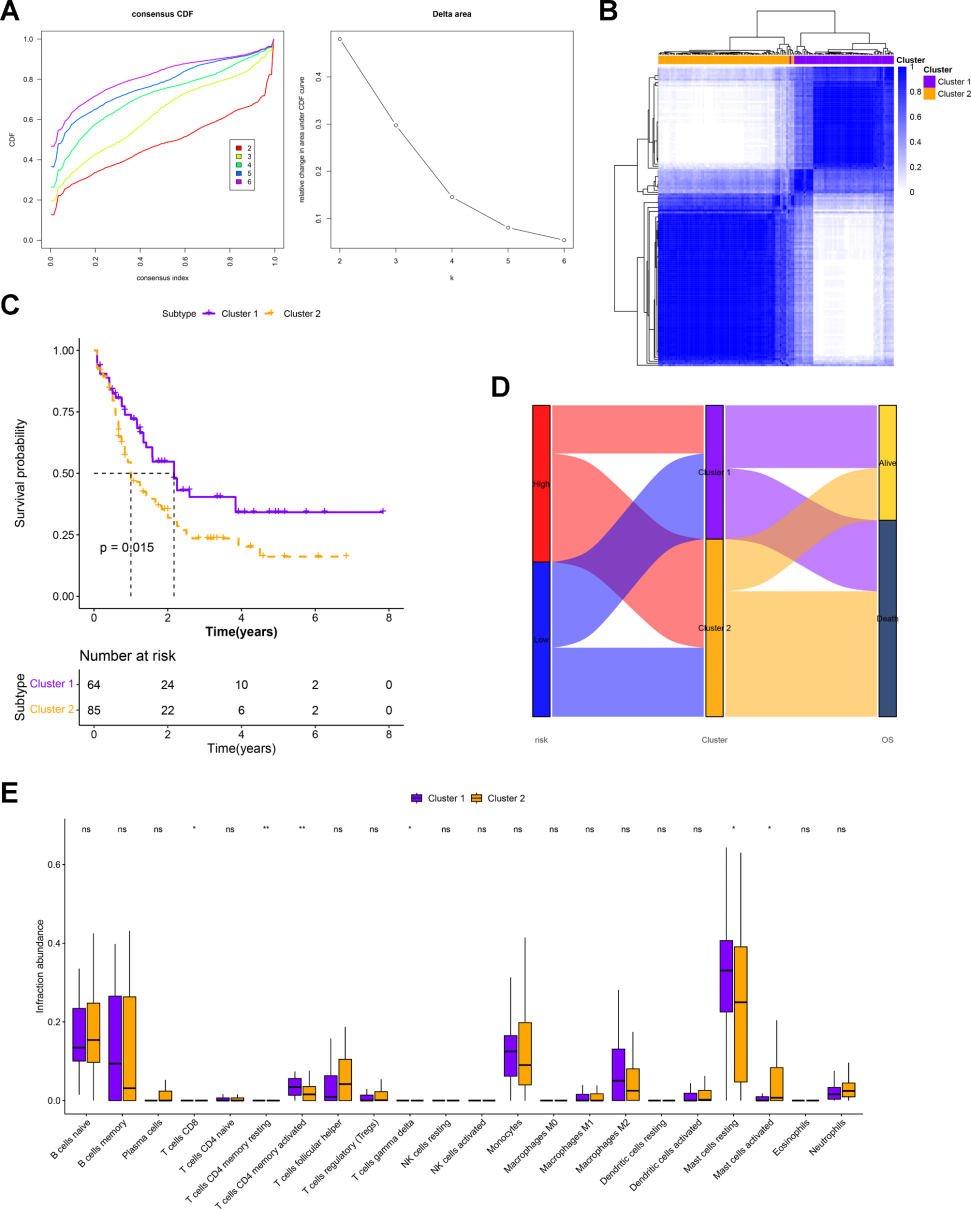
**Identification of two subtypes for AML and comparison between cluster 1 and 2**. **A**. Left panel: umulative distribution function (CDF) for consensus clustering with K = 2–6; Right panel: relative change in area under the CDF curve at K = 2–6. **B**. Consensus Clustering Matrix at Optimal K = 2. **C**. The KM curve showed the survival difference between the two clusters. **D**. Sankey diagram showed the distribution of samples with different prognostic risks between the two subgroups. **E**. Box plot showed the infiltration differences in the 22 types of immune cells between clusters 1 and 2. *p < 0.5; **p < 0.01

Unsupervised cluster analysis was performed based on the expression data of the eight prognostic signatures in the AML samples. By setting the range of K values from 2 to 6, the optimal K = 2 was selected (the curve was more stable) (Fig. 6A), and two clusters were obtained (Fig. 6B). By comparing the survival status between clusters 1 and 2, the KM curve revealed that patients in cluster 1 were prone to a favorable prognosis (Fig. 6C). The Sankey diagram also showed that patients who died were more distributed in Cluster 2, which comprised a greater proportion of patients with high prognostic risks (Fig. 6D). Using CIBERSORT, we identified six types of immune cells showing differences in infiltration between the two subtypes (Fig. 6E). Among them, CD8 T cells, γδT cells, and resting mast cells were significantly decreased in infiltration in cluster 2, which was more distributed in the high-risk group.

### Screening of hub genes using WGCNA

**Fig. 7:**
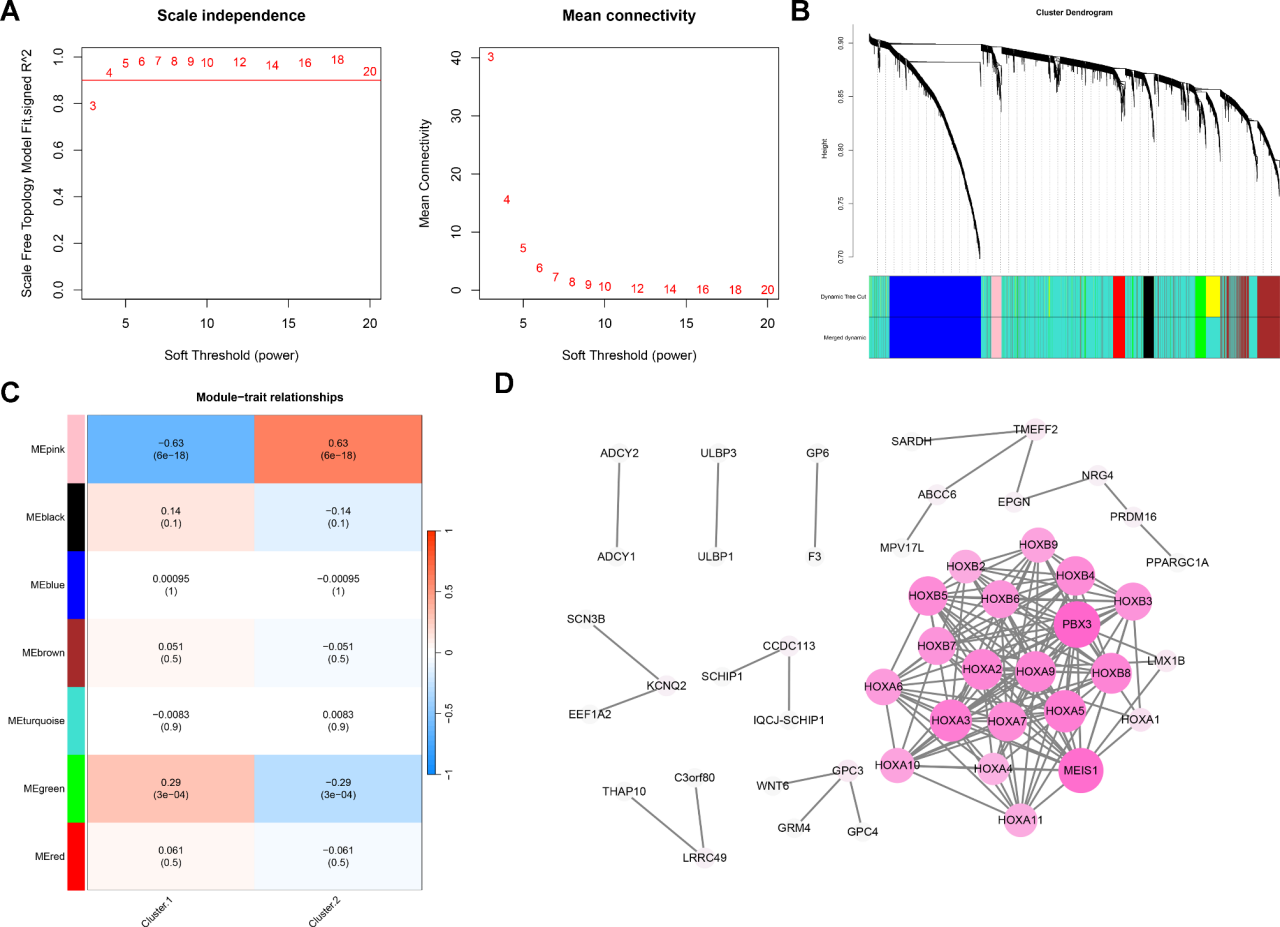
**Screening of hub genes related to AML subtypes using WGCNA and PPI analysis**. **A**. The scale-free fit index of β at soft thresholds of 1–20. **B**. The Genes were categorized into seven modules using hierarchical clustering. **C** Correlation between modules and subtypes. **D**. PPI networks comprising 48 genes and 161 interaction pairs

To screen for key genes further, we used subtype as a trait to construct a WGCNA network. When the power was 4, the network approximated a scale-free network distribution (Fig. 7A). Based on the dynamic tree-cutting algorithm, we set the minimum number of genes in each module to 30 and finally obtained seven modules (Fig. 7B). By calculating the relationships between the modules and subtypes, the pink module was found to have the strongest correlation with both subtypes (Fig. 7C). Next, 98 genes in the pink module were included in the PPI analysis. Based on the STRING database, a PPI network comprising 48 genes and 161 interaction pairs was constructed (Fig. 7D). Among them, the homeobox A cluster (*HOXA*) and homeobox B cluster (*HOXB*) family genes were considered to contribute more to AML subtypes because they had more degrees of connection in this PPI network.

## Discussion

Gln inhibition correlates with the level of glutaminase activity, and glutaminase inhibitors can suppress AML cell growth and induce cell apoptosis and differentiation of disease subtypes. Therefore, inhibition of Gln uptake is an attractive new strategy for treating AML [12, 22]. Key targets in the Gln metabolic pathway, such as proto-oncogenes, glutamic-pyruvic transaminase 2, and solute carrier family 1 member 5, are regulated by insulin-like growth factor 2 mRNA-binding protein 2 in an m6A-dependent manner to promote AML development and stem cell self-renewal [23]. Therefore, based on the 704 genes related to Gln metabolism, we screened 387 genes with significant differences in expression between AML and normal controls. Using univariate, LASSO, and stepwise Cox regression analyses, eight genes (*HGF*, *ANGPTL3*, *MB*, *F2*, *CALR*, *EIF4EBP1*, *EPHX1*, and *PDHA1*) were identified as a prognostic signature. The prognostic model constructed using the eight genes accurately predicted the prognostic risk in patients with AML, even in the validation cohort. Based on the expression levels of these genes, this study also categorized AML into two molecular subtypes, in which the prognostic risk of patients in cluster 2 was more adverse. Using the molecular subtype as a trait, this study also screened subtype-related module genes through WGCNA, among which the *HOXA* and *HOXB* family genes may be the key genetic features of disease subtypes. These results provided a valuable reference for further understanding the molecular mechanisms of these potential markers in AML and their impact on prognosis.

In the present study, we screened 387 DEGs associated with Gln expression in AML samples. Most somatic variations in these genes were missense mutations. Among these, the TOP10 genes with the highest mutation frequencies in AML were *IDH*, *TP53*, *WT1*, *IDH1*, *KRAS*, *PTPN11*, *ACACB*, *APC*, *NPC1*, and *QRICH2*. *IDH*, a mutated enzyme in the citric acid cycle, leads to the production of the oncogenic metabolite R-2-hydroxy-glutarate. This arrests the differentiation of hematopoietic stem cells, leading to the promotion of leukemia [24]. *TP53*-mutated AML is a unique subtype of AML with a poor prognosis [25]. A longitudinal study tracking the evolution of mutations demonstrated that *TP53* mutations represent primary mutational events in chemotherapy or radiation therapy-induced AML [26]. A meta-analysis suggested that *WT1* and TP53 mutations exhibit a mutually exclusive tendency in AML [27]. *WT1* has been reported to function as an oncogene and tumor suppressor in AML [28–30]. *IDH1* mutations occur in 6–10% of patients with AML [31], and inhibitors such as Ivosidenib and Azacitidine are currently available for this mutation [32]. A previous study found that *KRAS* mutations were associated with poor prognosis in AML [33]. Mutations in PTPN11 and *KRAS* confer resistance to combinations and multiple venetoclax combinations [34]. However, our understanding of the roles of *ACACB*, *APC*, *NPC1*, and *QRICH2* in AML is limited. This study identified mutated genes in AML that could contribute to treating AML.

Of the eight prognostic signatures identified in this study, seven genes, excluding *MB*, were significantly upregulated in AML. *HGF*, a hepatocyte growth factor, was confirmed to be upregulated in AML samples and cells and to promote the tumor malignancy of AML cells through targeted binding with miR-204 [35]. The mutation load of *CALR* alleles increases during the transformation from primary thrombocythemia to AML [36]. Activation of *EIF4EBP1*, eukaryotic translation initiation factor 4E binding protein 1, promotes AML cell proliferation and disease progression [37]. High expression of *EPHX1*, a microsomal epoxide hydrolase 1, is significantly associated with high recurrence and low overall survival rates in patients with AML [38], consistent with our findings, suggesting that *EPHX1* may promote disease progression in AML. *ANGPTL3* suppresses the expression of Ikaros, a key regulator of hematopoietic cell differentiation, thereby promoting the expansion and stemness of HSCs. It stimulates cancer growth by promoting angiogenesis, cell proliferation, and migration [39]. Recent studies have proposed that *PDHA1*, a key cuproptosis gene, is crucial for reprogramming glucose metabolism in tumor cells [40]. One study found that *PDHA1* was abnormally overexpressed in AML, consistent with our findings [41]. The core of thrombosis is thrombin, a product of F2, which participates in coagulation. The upregulation of *F2* may increase the risk of thrombotic bleeding complications in patients with AML [42]. *MB* transports and stores oxygen in muscle cells, and a decrease in *MB* in patients with AML may be related to impaired heart function [43]. The predictive effects of these eight genes on AML prognosis of AML is initially proposed in this study. A prognostic model constructed using eight genes accurately predicted prognostic risk in patients with AML.

Furthermore, this study found that the infiltration levels of CD8 T cells, γδT cells, resting NK cells, activated NK cells, and resting mast cells were significantly reduced in the high-risk prognostic group. Meanwhile, it is also found that the expression level of *EPHX1* was negatively correlated with CD8 T cells, γδT cells, resting NK cells, and activated NK cells, and the expression of *EIF4EBP1* was negatively correlated with CD8 T cells and γδT cells. Immune deficiency in AML is reflected in T cells and NK cells, where CD8 + T and diseased γδ T cells exhibit an exhausted state in AML at diagnosis [44]. However, the relationship of *EPHX1* and *EIF4EBP1* expression with these immune cells has not been investigated in AML. Therefore, this study proposed that *EIF4EBP1* and *EPHX1* contribute to immunodeficiency and further affect the prognostic survival status of AML patients by negatively modulating the infiltration level of CD8 T cells and γδT cells. However, this hypothesis requires further investigation.

Based on consistent cluster analysis, this study identified two molecular subtypes of AML, with cluster 2 tending to have a poorer prognosis. The samples in cluster 2 were also more distributed in the high-risk group. Furthermore, CD8 T cells, γδT cells, and resting mast cells, which were proportionately downregulated in the high-risk group, were similarly infiltrated in reduced abundance in cluster 2. These results suggested an increased prognostic risk for patients in cluster 2, suggesting that stratification based on disease subtypes can identify survival probability in AML. WGCNA further identified the module genes associated with the subtype, and the PPI network suggested that the *HOXA* and *HOXB* family genes may be key genetic characteristics of the disease subtype. Gene amplification, deep deletion, and alterations in the mRNA expression of *HOXA* have been found in approximately 18% of AML samples, and *HOXA3-10* serves as a potential AML therapeutic target and prognostic marker [45]. A relevant bioinformatics analysis revealed that six *HOXA* and three *HOXB* genes were significantly underexpressed and hypermethylated in AML, accompanied by favorable cytogenetic profiles [46]. Small-molecule inhibitors targeting the menin-lysine methyltransferase 2 A interaction restored normal *HOXA* expression in mutant AML, with therapeutic implications for AML patients [47]. In addition, *HOXA7*, *HOXA9*, and *HOXA11* are associated with AML risk status and prognosis [48]. To further support the above findings, this study suggested that *HOXA3-7*, *9–11*, and *HOXB2-9* expression were closely associated with the prognostic risk predicted based on eight prognostic signatures. The expression levels of these markers can help stratify patients with AML and predict their prognostic risks.

However, this study had some limitations. First, the predictive performances of these eight prognostic signatures must be independently validated using a cohort of clinical samples. Moreover, the relationship between these prognostic signatures and immune cell infiltration, as well as their potential regulatory mechanisms, needs to be explored through in vivo and in vitro experiments. In the future, we will continue to explore the potential of these eight genes as prognostic targets in AML and investigate the impact of their expression levels on the efficacy of immunotherapy.

## Conclusion

Based on 704 genes related to Gln metabolism, this study carried out differential expression analyses, as well as univariate, LASSO, and stepwise Cox regression analyses, and identified eight prognostic signatures. Prognostic and nomogram models constructed based on the expression levels can accurately identify the prognostic risk of AML. These prognostic signatures clustered patients with AML into two molecular subtypes with different prognostic risk patterns and immune profiles. Among the subtype-related module genes, the *HOXA* and *HOXB* family genes may be key genetic features of the AML subtypes.

## Data Availability

All data generated or analysed during this study are included in this article.

## Abbreviations

AML: Acute myeloid leukemia
Gln: glutamine
TCGA: the Cancer Genome Atlas
GTEx: Genotype-Tissue Expression
GEO: Gene Expression Omnibus
DEGs: differentially expressed genes
Gln-DEGs: Gln-related DEGs
KM: Kaplan-Meier
ROC: receiver operating characteristic
PPI: Protein-to-protein interaction

## References

[1] Shimony S, Stahl M, Stone RM. Acute myeloid leukemia: 2023 update on diagnosis, risk-stratification, and management. Am J Hematol. 2023;98(3):502–26. 36594187 10.1002/ajh.26822

[2] Pelcovits A, Niroula R. Acute myeloid leukemia: a review. Rhode Island Med J (2013). 2020;103(3):38–40. 32236160

[3] Kayser S, Levis MJ. The clinical impact of the molecular landscape of acute myeloid leukemia. Haematologica. 2023;108(2):308–20. 36722402 10.3324/haematol.2022.280801 PMC9890016

[4] Prada-Arismendy J, Arroyave JC, Röthlisberger S. Molecular biomarkers in acute myeloid leukemia. Blood Rev. 2017;31(1):63–76. 27639498 10.1016/j.blre.2016.08.005

[5] Stubbins RJ, Francis A, Kuchenbauer F, Sanford D. Management of Acute myeloid leukemia: a review for General practitioners in Oncology. Curr Oncol (Toronto Ont). 2022;29(9):6245–59. 10.3390/curroncol29090491 PMC9498246 36135060

[6] Weinberg OK, Porwit A, Orazi A, Hasserjian RP, Foucar K, Duncavage EJ, Arber DA. The International Consensus classification of acute myeloid leukemia. Virchows Archiv: Int J Pathol. 2023;482(1):27–37. 10.1007/s00428-022-03430-4 36264379

[7] Padmakumar D, Chandraprabha VR, Gopinath P, Vimala Devi ART, Anitha GRJ, Sreelatha MM, Padmakumar A, Sreedharan H. A concise review on the molecular genetics of acute myeloid leukemia. Leuk Res. 2021;111:106727. 34700049 10.1016/j.leukres.2021.106727

[8] Wojcicki AV, Kasowski MM, Sakamoto KM, Lacayo N. Metabolomics in acute myeloid leukemia. Mol Genet Metab. 2020;130(4):230–8. 32457018 10.1016/j.ymgme.2020.05.005

[9] Mishra SK, Millman SE, Zhang L. Metabolism in acute myeloid leukemia: mechanistic insights and therapeutic targets. Blood. 2023;141(10):1119–35. 36548959 10.1182/blood.2022018092 PMC10375271

[10] Kreitz J, Schönfeld C, Seibert M, Stolp V, Alshamleh I, Oellerich T, Steffen B, Schwalbe H, Schnütgen F, Kurrle N et al. Metabolic plasticity of Acute myeloid leukemia. Cells 2019, 8(8). 10.3390/cells8080805 PMC6721808 31370337

[11] Xiao Y, Hu B, Guo Y, Zhang D, Zhao Y, Chen Y, Li N, Yu L. Targeting glutamine metabolism as an attractive therapeutic strategy for Acute myeloid leukemia. Curr Treat Options Oncol. 2023;24(8):1021–35. 37249801 10.1007/s11864-023-01104-0 PMC10356674

[12] Willems L, Jacque N, Jacquel A, Neveux N, Maciel TT, Lambert M, Schmitt A, Poulain L, Green AS, Uzunov M, et al. Inhibiting glutamine uptake represents an attractive new strategy for treating acute myeloid leukemia. Blood. 2013;122(20):3521–32. 24014241 10.1182/blood-2013-03-493163 PMC3829119

[13] Tomczak K, Czerwińska P, Wiznerowicz M. The Cancer Genome Atlas (TCGA): an immeasurable source of knowledge. Contemp Oncol (Poznan Poland). 2015;19(1a):A68–77. 10.5114/wo.2014.47136 PMC4322527 25691825

[14] Barrett T, Wilhite SE, Ledoux P, Evangelista C, Kim IF, Tomashevsky M, Marshall KA, Phillippy KH, Sherman PM, Holko M, et al. NCBI GEO: archive for functional genomics data sets–update. Nucleic Acids Res. 2013;41(Database issue):D991–995. 23193258 10.1093/nar/gks1193 PMC3531084

[15] Ritchie ME, Phipson B, Wu D, Hu Y, Law CW, Shi W, Smyth GK. Limma powers differential expression analyses for RNA-sequencing and microarray studies. Nucleic Acids Res. 2015;43(7):e47. 25605792 10.1093/nar/gkv007 PMC4402510

[16] Engebretsen S, Bohlin J. Statistical predictions with glmnet. Clin Epigenetics. 2019;11(1):123. 31443682 10.1186/s13148-019-0730-1 PMC6708235

[17] Zhang Z, Geskus RB, Kattan MW, Zhang H, Liu T. Nomogram for survival analysis in the presence of competing risks. Annals Translational Med. 2017;5(20):403. 10.21037/atm.2017.07.27 PMC5673789 29152503

[18] Chen B, Khodadoust MS, Liu CL, Newman AM, Alizadeh AA. Profiling Tumor Infiltrating Immune Cells with CIBERSORT. *Methods in molecular biology (Clifton, NJ)* 2018, 1711:243–259. 10.1007/978-1-4939-7493-1_12 PMC5895181 29344893

[19] Wilkerson MD, Hayes DN. ConsensusClusterPlus: a class discovery tool with confidence assessments and item tracking. Bioinf (Oxford England). 2010;26(12):1572–3. 10.1093/bioinformatics/btq170 PMC2881355 20427518

[20] Langfelder P, Horvath S. WGCNA: an R package for weighted correlation network analysis. BMC Bioinformatics. 2008;9:559. 19114008 10.1186/1471-2105-9-559 PMC2631488

[21] Szklarczyk D, Gable AL, Lyon D, Junge A, Wyder S, Huerta-Cepas J, Simonovic M, Doncheva NT, Morris JH, Bork P, et al. STRING v11: protein-protein association networks with increased coverage, supporting functional discovery in genome-wide experimental datasets. Nucleic Acids Res. 2019;47(D1):D607–13. 30476243 10.1093/nar/gky1131 PMC6323986

[22] Matre P, Velez J, Jacamo R, Qi Y, Su X, Cai T, Chan SM, Lodi A, Sweeney SR, Ma H, et al. Inhibiting glutaminase in acute myeloid leukemia: metabolic dependency of selected AML subtypes. Oncotarget. 2016;7(48):79722–35. 27806325 10.18632/oncotarget.12944 PMC5340236

[23] Weng H, Huang F, Yu Z, Chen Z, Prince E, Kang Y, Zhou K, Li W, Hu J, Fu C, et al. The m(6)a reader IGF2BP2 regulates glutamine metabolism and represents a therapeutic target in acute myeloid leukemia. Cancer Cell. 2022;40(12):1566–e15821510. 36306790 10.1016/j.ccell.2022.10.004 PMC9772162

[24] Reed DR, Elsarrag RZ, Morris AL, Keng MK. Enasidenib in acute myeloid leukemia: clinical development and perspectives on treatment. Cancer Manage Res. 2019;11:8073–80. 10.2147/CMAR.S162784 PMC6724422 31564968

[25] Shin DY. TP53 mutation in Acute myeloid leukemia: An Old Foe Revisited. Cancers 2023, 15(19). 10.3390/cancers15194816 PMC10571655 37835510

[26] Smith SM, Le Beau MM, Huo D, Karrison T, Sobecks RM, Anastasi J, Vardiman JW, Rowley JD, Larson RA. Clinical-cytogenetic associations in 306 patients with therapy-related myelodysplasia and myeloid leukemia: the University of Chicago series. Blood. 2003;102(1):43–52. 12623843 10.1182/blood-2002-11-3343

[27] Yao Y, Chai X, Gong C, Zou L. WT1 inhibits AML cell proliferation in a p53-dependent manner. Cell Cycle (Georgetown Tex). 2021;20(16):1552–60. 34288813 10.1080/15384101.2021.1951938 PMC8409780

[28] Welch JS, Ley TJ, Link DC, Miller CA, Larson DE, Koboldt DC, Wartman LD, Lamprecht TL, Liu F, Xia J, et al. The origin and evolution of mutations in acute myeloid leukemia. Cell. 2012;150(2):264–78. 22817890 10.1016/j.cell.2012.06.023 PMC3407563

[29] Ley TJ, Miller C, Ding L, Raphael BJ, Mungall AJ, Robertson A, Hoadley K, Triche TJ Jr., Laird PW, Baty JD, et al. Genomic and epigenomic landscapes of adult de novo acute myeloid leukemia. N Engl J Med. 2013;368(22):2059–74. 23634996 10.1056/NEJMoa1301689 PMC3767041

[30] Hosen N, Shirakata T, Nishida S, Yanagihara M, Tsuboi A, Kawakami M, Oji Y, Oka Y, Okabe M, Tan B, et al. The Wilms’ tumor gene WT1-GFP knock-in mouse reveals the dynamic regulation of WT1 expression in normal and leukemic hematopoiesis. Leukemia. 2007;21(8):1783–91. 17525726 10.1038/sj.leu.2404752

[31] DiNardo CD, Stein EM, de Botton S, Roboz GJ, Altman JK, Mims AS, Swords R, Collins RH, Mannis GN, Pollyea DA, et al. Durable remissions with Ivosidenib in IDH1-Mutated relapsed or refractory AML. N Engl J Med. 2018;378(25):2386–98. 29860938 10.1056/NEJMoa1716984

[32] Gil-Sierra MD, Briceño-Casado MP, Sierra-Sanchez JF. Ivosidenib and Azacitidine in IDH1-Mutated AML. N Engl J Med. 2022;386(26):2535–6. 35767448 10.1056/NEJMc2206489

[33] Mustafa Ali MK, Williams MT, Corley EM, AlKaabba F, Niyongere S. Impact of KRAS and NRAS mutations on outcomes in acute myeloid leukemia. Leuk Lymphoma. 2023;64(5):962–71. 37042657 10.1080/10428194.2023.2190432

[34] Zhang H, Nakauchi Y, Köhnke T, Stafford M, Bottomly D, Thomas R, Wilmot B, McWeeney SK, Majeti R, Tyner JW. Integrated analysis of patient samples identifies biomarkers for venetoclax efficacy and combination strategies in acute myeloid leukemia. Nat cancer. 2020;1(8):826–39. 33123685 10.1038/s43018-020-0103-x PMC7591155

[35] Nie D, Ma P, Chen Y, Zhao H, Liu L, Xin D, Cao W, Wang F, Meng X, Liu L, et al. MiR-204 suppresses the progression of acute myeloid leukemia through HGF/c-Met pathway. Hematol (Amsterdam Netherlands). 2021;26(1):931–9. 10.1080/16078454.2021.1981533 34789086

[36] Langabeer SE, Haslam K, Elhassadi E. The mutant CALR allele burden in essential thrombocythemia at transformation to acute myeloid leukemia. Blood Cells Mol Dis. 2017;65:66–7. 28552475 10.1016/j.bcmd.2017.05.004

[37] Jiang Y, Wu SY, Chen YL, Zhang ZM, Tao YF, Xie Y, Liao XM, Li XL, Li G, Wu D, et al. CEBPG promotes acute myeloid leukemia progression by enhancing EIF4EBP1. Cancer Cell Int. 2021;21(1):598. 34743716 10.1186/s12935-021-02305-z PMC8574011

[38] Cheng H, Huang C, Tang G, Qiu H, Gao L, Zhang W, Wang J, Yang J, Chen L. Emerging role of EPHX1 in chemoresistance of acute myeloid leukemia by regurlating drug-metabolizing enzymes and apoptotic signaling. Mol Carcinog. 2019;58(5):808–19. 30644597 10.1002/mc.22973

[39] Jiang S, Qiu GH, Zhu N, Hu ZY, Liao DF, Qin L. ANGPTL3: a novel biomarker and promising therapeutic target. J Drug Target. 2019;27(8):876–84. 30615486 10.1080/1061186X.2019.1566342

[40] Deng L, Jiang A, Zeng H, Peng X, Song L. Comprehensive analyses of PDHA1 that serves as a predictive biomarker for immunotherapy response in cancer. Front Pharmacol. 2022;13:947372. 36003495 10.3389/fphar.2022.947372 PMC9393251

[41] Abulimiti M, Jia ZY, Wu Y, Yu J, Gong YH, Guan N, Xiong DQ, Ding N, Uddin N, Wang J. Exploring and clinical validation of prognostic significance and therapeutic implications of copper homeostasis-related gene dysregulation in acute myeloid leukemia. Ann Hematol. 2024;103(8):2797–826. 38879648 10.1007/s00277-024-05841-6

[42] Langer F, Quick H, Beitzen-Heineke A, Janjetovic S, Mäder J, Lehr C, Bokemeyer C, Kuta P, Renné T, Fiedler W, et al. Regulation of coagulation activation in newly diagnosed AML by the heme enzyme myeloperoxidase. Thromb Res. 2023;229:155–63. 37473552 10.1016/j.thromres.2023.07.006

[43] Specchia G, Buquicchio C, Pansini N, Di Serio F, Liso V, Pastore D, Greco G, Ciuffreda L, Mestice A, Liso A. Monitoring of cardiac function on the basis of serum troponin I levels in patients with acute leukemia treated with anthracyclines. J Lab Clin Med. 2005;145(4):212–20. 15962840 10.1016/j.lab.2005.02.003

[44] Tang L, Wu J, Li CG, Jiang HW, Xu M, Du M, Yin Z, Mei H, Hu Y. Characterization of Immune Dysfunction and Identification of Prognostic Immune-related risk factors in Acute myeloid leukemia. Clin cancer Research: Official J Am Association Cancer Res. 2020;26(7):1763–72. 10.1158/1078-0432.CCR-19-3003 31911547

[45] Reddel CJ, Tan CW, Chen VM. Thrombin Generation and Cancer: contributors and consequences. Cancers 2019, 11(1). 10.3390/cancers11010100 PMC6356447 30654498

[46] Wang H, Lin SY, Hu FF, Guo AY, Hu H. The expression and regulation of HOX genes and membrane proteins among different cytogenetic groups of acute myeloid leukemia. Mol Genet Genom Med. 2020;8(9):e1365. 10.1002/mgg3.1365 PMC7507697 32614525

[47] Juul-Dam KL, Shukla NN, Cooper TM, Cuglievan B, Heidenreich O, Kolb EA, Rasouli M, Hasle H, Zwaan CM. Therapeutic targeting in pediatric acute myeloid leukemia with aberrant HOX/MEIS1 expression. Eur J Med Genet. 2023;66(12):104869. 38174649 10.1016/j.ejmg.2023.104869 PMC11195042

[48] Xu P, Zhou D, Yan G, Ouyang J, Chen B. Correlation of miR-181a and three HOXA genes as useful biomarkers in acute myeloid leukemia. Int J Lab Hematol. 2020;42(1):16–22. 31670914 10.1111/ijlh.13116

